# Transcriptomic Analysis of the Acute Skeletal Muscle Effects after Intramuscular DNA Electroporation Reveals Inflammatory Signaling

**DOI:** 10.3390/vaccines10122037

**Published:** 2022-11-29

**Authors:** Amanda Sales Conniff, Jared Tur, Kristopher Kohena, Min Zhang, Justin Gibbons, Loree C. Heller

**Affiliations:** 1 Department of Medical Engineering, University of South Florida, Tampa, FL 33612, USA; 2 USF Genomics Core, University of South Florida, Tampa, FL 33612, USA; 3 USF Omics Hub, University of South Florida, Tampa, FL 33612, USA

**Keywords:** skeletal muscle, plasmid electroporation, RNA sequencing, signaling pathways

## Abstract

Skeletal muscle is a promising tissue for therapeutic gene delivery because it is highly vascularized, accessible, and capable of synthesizing protein for therapies or vaccines. The application of electric pulses (electroporation) enhances plasmid DNA delivery and expression by increasing membrane permeability. Four hours after plasmid electroporation, we evaluated acute gene and protein expression changes in mouse skeletal muscle to identify regulated genes and genetic pathways. RNA sequencing followed by functional annotation was used to evaluate differentially expressed mRNAs. Our data highlighted immune signaling pathways that may influence the effectiveness of DNA electroporation. Cytokine and chemokine protein levels in muscle lysates revealed the upregulation of a subset of inflammatory proteins and confirmed the RNA sequencing analysis. Several regulated DNA-specific pattern recognition receptor mRNAs were also detected. Identifying unique molecular changes in the muscle will facilitate a better understanding of the underlying molecular mechanisms and the development of safety biomarkers and novel strategies to improve skeletal muscle targeted gene therapy.

## 1. Introduction

Clinical application of nonviral gene therapies significantly advanced with the development of the lipid nanoparticle—modified mRNA COVID vaccines [1,2]. Lipid nanoparticles primarily deliver their nucleic acid cargo via endocytic mechanisms [3]. This is also true of other nonviral delivery methods such as electroporation, which delivers nucleic acids into cells by endocytosis-like mechanisms; caveolin/raft-mediated endocytosis, clathrin-mediated endocytosis, and micropinocytosis have been proposed [4,5]. In this study, we focus on the acute gene and protein expression changes in immune signaling pathways in response to intramuscular plasmid electroporation.

Electroporation has been used widely among non-viral gene transfer techniques to introduce DNA and other molecules into cells in vitro and in vivo in a simple, inexpensive, and safe manner. The relatively low transfection efficiency of plasmid DNA (pDNA) in tissues including skeletal muscle tissue is increased by applying electric pulses [6]. Therefore, plasmid electroporation becomes an excellent tool for delivery of therapeutic proteins [7] and vaccines [8].

Early large animal studies of intramuscular pDNA electrotransfer including hemophilia gene therapy dogs [9], viral vaccines in pigs [10] and subsequent studies predicted that this technique could be translated to use in veterinary and clinical studies. This technique has been used in veterinary practice for cancer vaccines [11,12]. Intramuscular electroporation for plasmid delivery has also been tested in several recent clinical trials. In the initial cancer gene therapy Phase 1 trial, patients were treated with electroporation of a plasmid encoding prostate-specific membrane antigen, which generated a persistent antibody response [13]. Additional trials have targeted muscle for expression of an integrin inhibiting peptide [14] and a vaccine to telomerase reverse transcriptase as a tumor antigen in a prime-boost strategy with adenoviral delivery [15] An early vaccine clinical study targeted Hepatitis C virus [16]. Subsequent trials have supported the safety and potential impact of intramuscular pDNA electroporation [8]. In each trial, the therapy was well-tolerated, which was in agreement with two plasmid delivery-based vaccine safety trials which found that injection site reactions that resolved within a few minutes were the most common adverse event [17,18].

Although DNA electrotransfer has been widely studied in vitro and in vivo environments, the acute molecular effects of plasmid DNA delivery through cells and tissue are rarely characterized. An early study of intramuscular DNA electroporation in pigs demonstrated that IM GET significantly enhanced antigen expression and that the vaccine effect is potentiated by the accompanying inflammation [19]. Here, we used RNA sequencing (RNA-seq) analysis of the muscle to clarify specific signatures of pDNA electrotransfer, providing information on the molecular changes after the procedure. We compared differentially regulated genes related to inflammatory pathways among groups receiving muscle pDNA injection, pulse application, as well as the combination, identifying unique and shared molecular changes.

## 2. Materials and Methods

### 2.1. Animals and Plasmid DNA Delivery

All procedures were approved by the University of South Florida Institutional Animal Care and Use Committee (protocol R IS00007249, 2019). Female 7 to 8 week female C57Bl/6J mice (Jackson Laboratories, Bar Harbor, ME, USA) were used for this study. For all procedures, the animals were anesthetized in an induction chamber infused with 2.5% isoflurane (Mallinckrodt Veterinary Inc., Mundelein, IL, USA) in O2 and fitted with a standard rodent mask supplied with the same mixture. The empty vector plasmid gWiz-blank was commercially prepared (Aldevron, Fargo, ND, USA) and suspended to 2 mg/mL in physiological saline. Untreated muscle was used as a control group while the experimental groups were intramuscular pDNA injection alone, saline injection followed by pulses, and pDNA injection followed by pulses. Fifty μL of pDNA (gWiz-blank) or saline was injected into the right caudal thigh muscle. A 2-needle electrode with a 5 mm gap (BTX Harvard Apparatus, Holliston, MA, USA) was placed into the muscle surrounding the injection site and eight 20 ms pulses at a voltage to distance ratio of 100 V/cm were immediately applied using an ECM830 pulse generator (BTX). Each mouse was monitored continuously until recovery from anesthesia. After four hours, the animals were euthanized, the muscle samples were collected and snap frozen on dry ice.

### 2.2. Total RNA Extraction, Library Preparation and RNA Sequencing

Muscle samples were homogenized in Ribozol (VWR Life Sciences, Radnor, PA, USA) using a gentle Macs Octo Dissociator (Miltenyi Biotec, Bergisch Gladbach, Germany) and purified using RNA Clean and Concentrator kits (Zymo Research, Irvine, CA, USA) per manufacturer’s instructions. One μg of total RNA samples was used for the sequencing, RNA quantification by Qubit (Thermo Fisher Scientific, Waltham, MA, USA) and sample quality checks by TapeStation (Agilent Technologies, Santa Clara, CA, USA). Three of the 20 samples had RNA integrity number lower than 8.0; these samples were eliminated from the study; therefore, sample replicates were 4–5 per group. We used the Illumina TruSeq stranded mRNA kit (Cat. 20020594) with the single indexes kit (Cat. 20020492, 2002093) for the library preparation. RNA sequencing was performed using the Illumina NextSeq 550 system (Illumina, Carlsbad, CA, USA), and high output reagent kit 150 cycles. Total output was 20 million paired-end reads per sample. The quality of the reads was assessed using FastQC version 0.10.1 (Babraham Institute, Cambridge, UK), which indicated no sequence trimming was necessary. The sequences were directly aligned to the Mus musculus reference genome assembly (GRCm38.p6) using the R packages HISAT2 version 2.1.0 (Center for Computational Biology, McKusick-Nathans Institute of Genetic Medicine, Johns Hopkins University, Baltimore, Maryland, USA) for alignment and Samtools version 1.3.1 (Wellcome Trust Sanger Institute, Cambridge, UK and Broad Institute of MIT and Harvard, Cambridge, MA, USA) for sorting and conversion to bam files. Read counts for gene regions were obtained with FeatureCounts from Subread version 1.6.3 using Ensembl annotations GRCm38.99 GFF.

Prior to examining gene expression, raw read counts were normalized using DESeq2 [20] to correct for reading count differences caused by differences in composition and sequencing depth between the samples. After normalization, statistical evaluation was performed to identify differentially expressed genes in pairwise comparisons using DESeq2. Differential gene set expression analysis focusing on immunogenic pathways was assigned via a Kyoto Encyclopedia of Genes and Genomes (KEGG) [21,22,23,24] accession number that was performed using the R package GSAR [25]. A gene set was considered differentially expressed if the false discovery rate was less than or equal to 0.1. The results were graphed using the R package ggplot2 [26]. Significance between groups was determined by a one-way ANOVA test followed by a Tukey–Kramer post-test using GraphPad InStat (San Diego, CA, USA). A *p* < 0.05 was considered statistically significant. Gene symbols in text are per the Mouse Genome Database [27].

### 2.3. Protein Quantification by Bead Array

Muscle tissue was extracted and lysed using Mammalian Protein Extraction Buffer (GE Healthcare, Milwaukee, WI, USA). The total protein concentration in the tissue lysate was normalized and 25 µg of each sample was analyzed using a premixed multiplex panel (Mouse Cytokine/Chemokine Magnetic Luminex Assay, Millipore, Burlington, MA, USA) on a MAGPIX System (Luminex, Austin, TX, USA) per manufacturer’s instructions. All protein identifiers are per Uniprot [28].

## 3. Results

We used heatmaps to identify the biological interpretation of our significant differentially expressed gene sets. The gene expression of the differentially expressed gene sets was clustered for visualization. The data shows the identification of genes commonly regulated between the groups. The color intensity of the boxes was based on the z-score normalization, which indicates how many standard deviations a value falls above or below the mean.

### 3.1. Genome-Wide Expression Analysis

**Figure 1:**
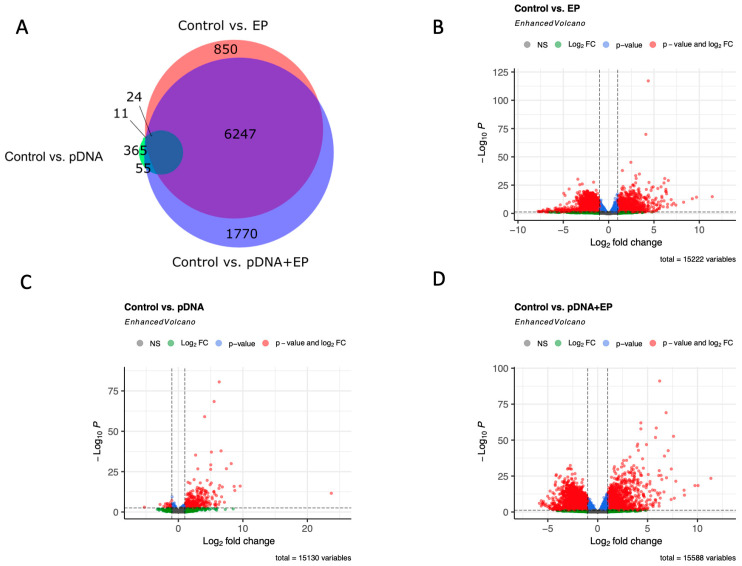
Differential expression analysis between groups. Differentially expressed genes with adjusted *p*-values < 0.001 and an absolute log2 fold changes > 2 were considered significant. (**A**) Venn diagram shows the distribution of differentially expressed among pairwise comparisons. Volcano plots show significant gene expression changes between control skeletal muscle and (**B**) pulsed muscle, (**C**) muscle receiving plasmid injection only, and (**D**) muscle receiving plasmid electroporation.

Genome-wide expression analysis was performed to identify genes regulated by pDNA electroporation. An analysis of the transcriptome (Figure 1A) revealed that injection of DNA to the muscle generated the fewest differentially expressed genes (DEGs), 365, when compared to control muscle. Applying pulses to muscle generated 850 DEGs, while the combination generated 1770 DEGs with respect to the control. All comparisons found a total of 6247 genes to be differentially expressed. Volcano plots are shown to provide an overview of the differential expression of genes between control caudal thigh muscle and pulsed muscle (Figure 1B), muscle injected with pDNA (Figure 1C), and muscle subjected to pDNA electroporation (Figure 1C).

### 3.2. Chemokine mRNA Expression Is Regulated in Mouse Muscle after Plasmid Electroporation

**Figure 2:**
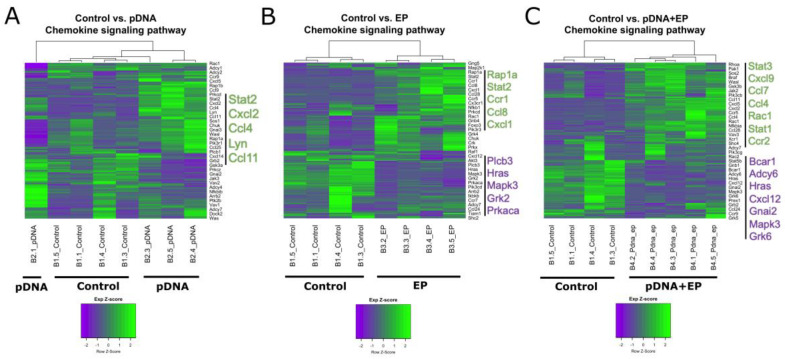
Heatmap gene cluster grouping for control and experimental groups for the chemokine signaling pathway. (**A**) pDNA, gWiz-blank injection, (**B**) EP, electroporation, and (**C**) pDNA + EP, gWiz-blank injection followed by electroporation. The dendrograms depicting hierarchical clustering are based on the expression of genes in the given set. The columns are samples and the rows are genes. Green represents above average expression while purple represents below average expression. Specific up-regulated and down-regulated genes are emphasized in green or purple font, respectively. n = 4–5 per group.

Several immune signaling pathways were regulated after pDNA electroporation. In many ways, these pathways overlap in function. The chemokine signaling pathway was one of the most upregulated KEGG pathways [21,23,24] (Figure 2). Although several chemokine ligands and receptor mRNAs were upregulated in each experimental group compared to the control group, there was little consistency between the groups, with the exception of C-C motif chemokine 4 (Ccl4). This mRNA was upregulated after pDNA injection regardless of pulse application.

### 3.3. Chemokine Proteins Are Regulated in Mouse Muscle after Plasmid Electroporation

**Figure 3:**
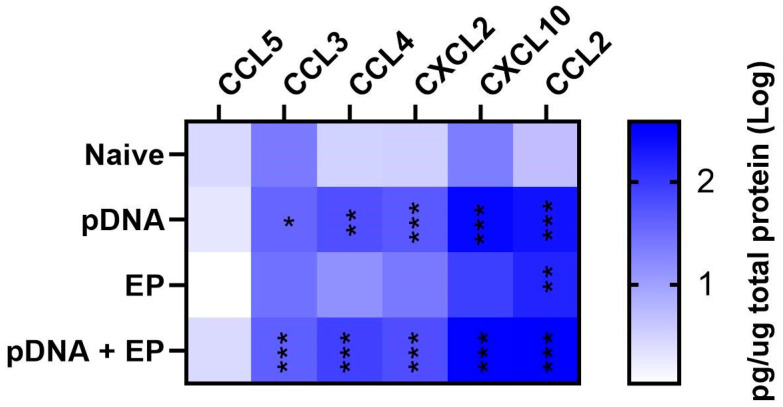
Chemokine expression in mouse skeletal muscle four hours after plasmid delivery. *** *p* < 0.001, ** *p* < 0.01, * *p* < 0.05 compared to control. n = 4−5 per group.

We next quantified several chemokine proteins four hours after pDNA injection, pulse application, or the combination using a bead array (Figure 3). CCL2, CCL3, CCL4, C-X-C motif chemokine 2 (CXCL2) and CXCL10 protein levels increased in groups receiving DNA injection, although electroporation also contributed to CCL2 expression and significantly increased its production (*p* < 0.01). CCL5 was not regulated in any group. The primary driver of chemokine expression was plasmid injection.

### 3.4. Putative DNA-Specific Pattern Recognition Receptor (PRR) mRNAs Are Regulated

Plasmid DNA is produced in bacteria and constitutes a pathogen association molecular pattern (PAMP). PAMPs are detected by pattern recognition receptors (PRRs), setting off a signaling cascade culminating inflammatory signaling [29,30,31]. Many putative DNA-specific PRRs have been described, and activation of these receptors generates the production of cytokines, chemokines and Type I interferons. Table 1 shows putative PRRs that are regulated at the mRNA level four hours after pDNA injection, pulse application, or the combination with a significant absolute log2 fold change >2. Zbp1, Ifi204, and Cgas mRNA expression was elevated in all experimental groups when compared to the control group. No significant differences in gene expression were detected between the experimental groups for Zbp1, Ifi204, Cgas and Ddx60. The mRNAs of helicases Ddx58 and Ddx60 were significantly upregulated in muscles into which pDNA was injected.

## 4. Discussion

It is well established that intramuscular injection of plasmid DNA causes an inflammatory response and the recruitment of macrophages and T lymphocytes [39]. Chemokines are not only important chemoattractants, but expression is also associated with muscle repair and regeneration [40]. In this study, the chemokine mRNA and protein expression detected was primarily in response to pDNA injection with or without pulsing. Therefore, the primary regulator of chemokine signaling was likely plasmid DNA. Chemokine expression is a driver of the inflammatory response typically associated with the ligands of receptors CCR1, CCR2 and CXCR3 among others [41]. We detected increase production of CCR1 ligands CCL3 and CCL4 in both groups receiving pDNA injection as well as CXCL10, a CXCR3 ligand, in the pDNA electroporation group. It is difficult to explain why CXCL12, a CXCR4 ligand, was upregulated after pDNA injection but downregulated after pDNA electroporation. Uniquely, CCL2 protein, a CCR2 ligand, was upregulated by any tissue manipulation. This regulation may be driven by plasmid DNA exposure or by the mechanical injury of muscle injection or pulse application. CCL2 is an inflammatory mediator that may play a regenerative role in the recovery to muscle injury [42,43].

Chemokine expression may be driven by intracellular sensing of plasmid DNA [44]. Around 20 putative DNA-specific PRRs have been described. These PRRs may be activated sequentially to escalate or control inflammatory responses [45,46]. These responses may also be controlled by interplay between PRR signaling pathways [45,46]. We previously established that many cell types [47,48,49], including myoblasts [50], and tissues [47,51,52] respond to nucleic acid electroporation by the production of immune modulators. In myoblasts, Zbp1, Ifi204, and Ddx60 mRNAs are regulated in cells receiving pDNA electroporation, while Cgas mRNA is unregulated [50]. Caudal thigh muscle data confirm this observation, but mRNA regulation is also observed in response to simple pDNA injections. This could be due to the response of other cell types such as satellite cells [53].

Several previous studies have addressed gene expression after intramuscular pDNA electroporation. A similar study using both chemokine and stress arrays, pulse application alone induced short-term chemokine expression along with a number of stress mRNAs; a combination with pDNA was not tested [54]. Although no direct overlap in specific mRNAs was observed, we did observe chemokine mRNA and protein regulation in response to pulse application. The induction of chemokine expression may be more significant because the pulses used in the earlier study were much longer and at a higher voltage to distance ratio. While a number of pathways were regulated, another study found a limited inflammatory response [55]. The investigators injected less DNA, the primary driver of inflammation in this study, which may explain this discrepancy. The inconsistencies between these two studies may also be due to the different pulse regimens used and to perturbations of cDNA levels due to the second-strand cDNA synthesis method used in the earlier study. In many aspects, our results confirm or parallel a previous study of immune and tissue responses seven days after intramuscular pDNA electroporation [56]. We confirmed the observation of Mann et al. of increased levels of CXCL9 mRNA in response to pDNA electrotransfer, but not pulses alone. However, we did not confirm changes in CCL5 mRNA levels. Our results also diverged on the precise factors regulating of the mRNA levels of the PRR ZBP1. Although the experimental design of the two studies had many differences, a potential driver of these differences was the time points investigated. This study focused on acute after four hours, while Mann et al. focused on more chronic effects of pDNA electroporation. Overall, the studies varied in mouse model and sex, specific muscle targeted, plasmid composition, quantity and volume, pulse protocols applied, electrodes used for delivery, time points assayed, and assay method among other differences, so incongruities between this study and previous studies may be due to differences in experimental design.

Four hours after pDNA electroporation, some genes and proteins, including specific chemokines and DNA binding proteins, are regulated in response to pDNA injection and therefore potentially related to PRR activation. However, skeletal muscle is capable of regeneration after exercise or injury [57]; regulation may also be related to muscle regeneration from the insults of pDNA injection, pulse application or the combination.

Understanding which genes are perturbed in the electroporation of pDNA to the skeletal muscle could pave the way for identifying markers as potential predictors of inflammation and can be validated as possible therapeutic targets. Additionally, the discovery of potential therapies may be enabled by high-throughput systems using biological analyses, particularly at the transcriptome levels.

Innate immune responses are important to vaccine efficacy [58,59]. PRR agonists such as nucleic acids induce innate immunity to act as vaccine adjuvants [60,61]. Proinflammatory chemokine production is important to this process for the recruitment of a variety of immune cells to the vaccine site [61,62]. Here, we demonstrate that both putative DNA-specific PRRs and proinflammatory chemokines are regulated after intramuscular DNA electroporation, potentially contributing to vaccine efficacy.

## Figures and Tables

**Table 1:** Fold changes in mRNA levels of putative DNA-specific PRRs. Greater than 2 log2 regulation (*, *p* < 0.05; **, *p* < 0.01; ***, *p* < 0.001 with respect to the control group).

PRR	Reference	pDNA	EP	pDNA + EP
Z-DNA binding protein 1 (Zbp1/Dai/Dlm)	[32]	2.96 ***	2.73 **	3.04 ***
Retinoic acid inducible gene I (RIG-I/Ddx58)	[33,34,35]	1.97 ***	0.87	2.05 ***
Interferon-activable protein 204 (Ifi204)	[36]	3.13 ***	3.55 ***	3.92 ***
DEAD (Asp-Glu-Ala-Asp) box polypeptide 60 (Ddx60)	[37]	1.92 *	1.31	2.01 **
Cyclic guanosine monophosphate adenosine monophosphate synthase (Cgas)	[38]	1.72 *	1.58 *	2.43 ***

PRR, pattern recognition receptor, pDNA, plasmid DNA; EP, electroporation.

## Data Availability

The data presented in this study are openly available in Mendeley Data, V1, https://doi.org/10.5038/f73phw648m.1 (accessed on 25 November 2022).
